# A Century of Radiation Therapy and Adaptive Immunity

**DOI:** 10.3389/fimmu.2017.00431

**Published:** 2017-04-11

**Authors:** Dörthe Schaue

**Affiliations:** ^1^Department of Radiation Oncology, David Geffen School of Medicine, University of California at Los Angeles, Los Angeles, CA, USA

**Keywords:** radiation, tumor immunity, inflammation, lymphocytes, tolerance

## Abstract

The coming of age for immunotherapy (IT) as a genuine treatment option for cancer patients through the development of new and effective agents, in particular immune checkpoint inhibitors, has led to a huge renaissance of an old idea, namely to harness the power of the immune system to that of radiation therapy (RT). It is not an overstatement to say that the combination of RT with IT has provided a new conceptual platform that has re-energized the field of radiation oncology as a whole. One only has to look at the immense rise in sessions at professional conferences and in grant applications dealing with this topic to see its emergence as a force, while the number of published reviews on the topic is staggering. At the time of writing, over 97 clinical trials have been registered using checkpoint inhibitors with RT to treat almost 7,000 patients, driven in part by strong competition between pharmaceutical products eager to find their market niche. Yet, for the most part, this enthusiasm is based on relatively limited recent data, and on the clinical success of immune checkpoint inhibitors as single agents. A few preclinical studies on RT–IT combinations have added real value to our understanding of these complex interactions, but many assumptions remain. It seems therefore appropriate to go back in time and pull together what actually has been a long history of investigations into radiation and the immune system (Figure 1) in an effort to provide context for this interesting combination of cancer therapies.

Those who cannot remember the past are condemned to repeat it.

George Santayana

## Dedication

A scientific journey dedicated to William H. McBride for his contributions to the field.

## Radiation is Handed Out, Immune Cells Come In

On December 29th in 1917 in a speech to the American Association for the Advancement of Science, Dr. James Ewing described in detail the effects of radium therapy in cancer (1). Using cervical cancer as an example, he noted an exudation of polymorphonuclear leukocytes and lymphocytes within 3–5 days of treatment, only to be followed later by plasma cell development and the formation of granulation tissue. Importantly, he suggested that it might be exactly this immune involvement that is essential for both tumor eradication and tissue healing (1).

One of the first scientists to firmly recognize that radiation modulates immunity was James Bumgardner Murphy (1884–1950). His large body of work performed at the Rockefeller Institute about 100 years ago focused on the role of lymphocytes in graft and tumor rejection and led to some truly innovative concepts and discoveries that have not received worthy recognition (2) (Forsduke).^1^ Murphy’s observations in mouse models led him to suggest that, “in the lymphoid elements we have an important link in the process of so-called cancer immunity.” He proposed that radiation can achieve immune stimulation and tumor protection in mice, depending on the radiation dose (extent of erythema), volume and site, and the time between exposure and tumor challenge (3–5). Russ et al. (6) looked into the effect of small doses of X-rays on blood white cell counts and on the resistance of rats to transplanted tumors. Their data and Murphy’s data concluded that X-rays, apart from their direct action on tissue cells have two indirect actions: “(a) large doses of X-rays, by destroying the immune conditions, will favour the growth of tumours, and (b) small doses, by producing immune conditions, will help to overcome the tumour.” A critical conclusion at that time was that “the therapeutic action of X-ray in cancer depended on the cellular reaction induced in the normal tissues surrounding the growth,” in particular the fact that radiation had the ability to switch a predominantly polymorphic infiltrate to a lymphoid one within a matter of days and that this was necessary for tumor rejection (7). Murphy further commented that “the lymphocyte is greatly affected by X-rays, since it is possible either to stimulate by small doses the production of these cells or by larger ones practically to destroy all the lymphoid tissues of the body” and by extension prevent tumor immune rejection. The cut-off was estimated to be around a mild erythema dose, which was the way dosimetry was performed in those days, i.e., around the time when orthovoltage machines were being introduced and dose delivery was limited to superficial depth. This is about 6–8 Gy, remarkably close to what is now widely (perhaps not incidentally) being considered as the preferred dose for hypofractionated radiotherapy either when used alone or in combination with immune intervention strategies (8–10). To put this in a broader context, this was also the time of the discovery of induced mutations and radiation carcinogenesis, generally ascribed to Muller in 1927 (11), which provided the impetus for the development of inbred mouse strains and a hugely important point of divergence of models for cancer immunology from those of graft rejection and the discovery of major histocompatibility complex (MHC) antigens. In fact, the Jackson Laboratories (Bar Harbor, ME, USA)^2^ was founded as an institution for “research in cancer and the effects of radiation” in 1929 by a geneticist named Clarence Cook Little (1888–1971) who aimed to develop genetically inbred mice that also paved the way for the radiation genetics “mega-mouse project” at Oak Ridge National Laboratories in Tennessee by Russell (12). Murphy’s studies took place largely before that and the models that he used, i.e., the white mice, were not completely syngeneic and as such not ideal for tumor transplantation because of graft rejection issues (13). He did however look into spontaneous as well as transplanted tumors and the thought processes still have great relevance for the field of Radiation Oncology today.

## Early Attempts at Combining Radiation Therapy (RT) with Immunotherapy (IT)

**Figure 1:**
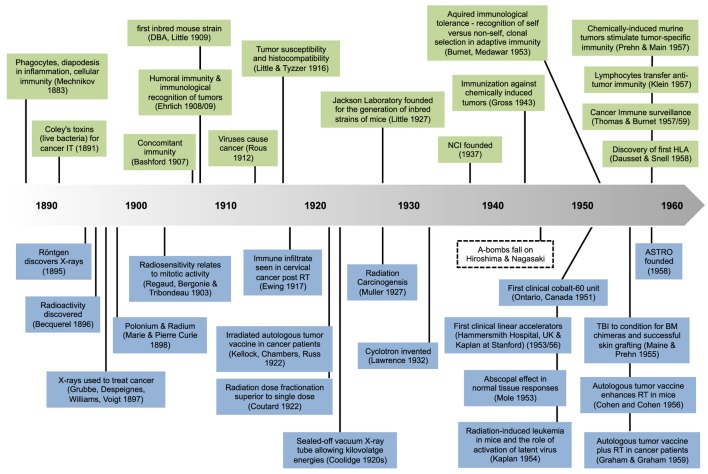

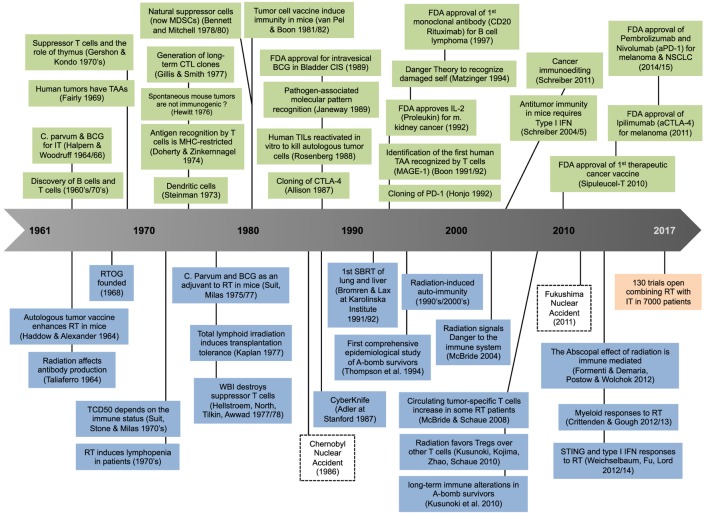
**Milestones in immunology (top) and radiation science (bottom)**.

The first attempts at combining IT and RT in mice and rats were probably from Cohen and Cohen in 1956/1960, followed by Sir Alexander Haddow and Sir Peter Alexander in 1964 (14–16) (Figure 1). Haddow contributed massively to the field of chemical carcinogenesis, while Alexander was the first immunologist to head a radiobiology lab and has published a popular book on “Atomic Radiation and Life” (17). Essentially, the Cohens, Haddow, and Alexander were able to show that the success of RT delivered to a murine mammary carcinoma (probably virus-induced) or a chemically induced (benzpyrene) fibrosarcoma could be substantially enhanced if it was preceded by a personalized vaccine. This involved taking tumor biopsies, irradiating them *ex vivo*, and injecting them back into the same animal prior to delivering *in vivo* radiation to the primary tumor. This basically acknowledged that tumor antigens were largely unique to each tumor. Vaccination before RT seemed more effective than the alternative sequence and better than vaccination alone as had been attempted in humans 40 years previously by Kellock et al. (18). Post-surgery, they had placed 2 rads-irradiated, minced autografts into 2 abdominal wall pockets of 30 late-stage cancer patients, mostly women with breast cancer, in an attempt to immunize them. Considering that they were dealing with late-stage disease, that the immunogenicity of the tumors was unknown and the absence of additional treatment (apart from one case who got RT), it is not surprising that the results were not as inspiring as the animal data mentioned above. More encouraging in this regard was a study on 101 patients also with advanced cancers, unfavorable prognosis and mostly of gynecologic origin where vaccination with autologous tumor cells in Freund’s adjuvant seemed able to improve responses to subsequent RT, at least in some patients (19).

The end of the 1960 and into the 1970s saw a resurgence of interest in IT led by the French and Scots. The approach was based on using bacteria in the hope to boost the immune system. Originally pioneered by Coley in 1891 (20), “Coley’s toxins” were utilized up until the early 1960s as a form of IT for cancer. Halpern and Woodruff chose *Corynebacterium parvum* (now *P. acnes*) or *bacillus Calmette–Guérin* (BCG) for the same purpose (21–23) and radiation biologists started to interrogate the potential of this form of IT as an adjuvant to RT (24–26). The conclusions were that *C. parvum* was especially beneficial to RT outcome (a) when given before rather than after local RT, (b) when radiation doses were small, and (c) when the tumor was intrinsically immunogenic. The tumor regression seen in the context *C. parvum* was largely based on the intense proliferation in lymphoreticuloendothelial tissues (spleen, liver, and lungs) and enhanced T cell activation, although stimulation of cytotoxic/cytostatic macrophages also contributed (27). Whether these *C. parvum*-primed T cells and macrophages were at play in a cooperative or rather a mutually exclusive fashion may have depended on the context (tumor or healthy) and the route of administration (28). BCG also appeared to boost the response of preclinical mammary tumors to RT (29), but the lack of cures seen following monotherapy with BCG or C. *parvum* in the clinic led to the demise of this form of IT. Nonetheless, to this day BCG remains the main intravesical IT for treating early-stage bladder cancer. Attempts to develop cancer vaccines continued throughout the rest of the twentieth century, with sporadic successes in individual patients, but without generating much overall enthusiasm for IT as a cancer therapy, and with few serious attempts to combine IT and RT.

## Lymphocyte Responses in the Irradiated Host—Dualism at Its Best

One can’t help but feeling that the field of natural immunity, as discovered by Ilya Mechnikov^3^ at the end of the nineteenth century, was somewhat overshadowed by the study of adaptive, antigen-specific immunity. For instance, the 1960s and 1970s was clearly the age of the lymphocyte. Along with the distinction between B and T lymphocyte lineages came the definition of MHC antigens and their role in directing T cell and B cell responses, and the role of the thymus in T cell development and tolerance (30, 31). This was further aided by improvements in lymphocyte culture and assays detecting their anti-cancer function both *in vitro* and *in vivo*. It is perhaps not surprising then that studies on radiation effects and immunity mirrored those in emphasis and more evidence as to the confusing duality of radiation effects started to accumulate. For instance, in 1964, Taliaferro et al. produced a monograph summarizing findings on radiation-induced modification of the antibody response (32). They noted that radiation can inhibit or enhance antibody formation and increase or decrease susceptibility to infections, depending on its nature. The authors pointed to evidence collected prior to 1950 that an antibody response tends to be much more effectively suppressed if the antigen is given *after* whole body irradiation (WBI) rather than if given *before*. This timing issue is of relevance today and it seems that an activated or memory immune system is more radioresistant than a naïve one. Importantly, they noted that “enhanced antibody production can be elicited in a radiation-damaged host provided the antigen is introduced at critical times” or if doses are small (about 100–200 rad WBI), echoing the early findings in cancer models mentioned earlier.

The early 1970s were marked by a focus on RT-induced lymphopenia in patients with breast, cervical, and bladder cancer (33–36). This was linked to various preclinical studies showing WBI or wide-field RT could enhance metastasis and the growth of immunogenic tumors outside the radiation field (37). Similarly, Kaplan and Murphy had reported in 1949 that suboptimal (400–1,000 rad) local tumor irradiation of a spontaneous mammary carcinoma in C57Bl/6 mice enhanced metastasis fourfold (38). On the other hand, as Essen pointed out in his review “virtually every modality employed in the treatment of cancer has demonstrated an adverse effect upon metastasis under some conditions,” so radiation was not unique in this respect (39). In fact, in most cases there was little evidence for immune involvement in causing this. Non-curative RT may be an exception, but in general distant metastases and radiocurability of the primary tumor do not seem linked (40).

The concept that prolonged RT-induced lymphocyte nadirs are generally associated with poor outcome is however valid—something that has recently gained renewed attention by Radiation Oncologists. In the 1970s, it was already apparent that the tissue, the size of the field, the delivery schedule, and the dose were important factors in determining the extent of lymphodepletion (41). Even today, in spite of superior computer-aided delivery systems and smaller high dose fields, a significant drop in circulating lymphocytes remains a reality for most irradiated patients. Since lymphocytes are very radiosensitive, dose is of less importance than field volume and hypofractionation generally spares these cells by limiting time, i.e., volume blood passing through, compared to a conventional 6-week delivery. On the other hand, intensity-modulated radiotherapy (IMRT) may on occasion have the opposite effect because the whole body dose can be large. Our current picture is made somewhat more sophisticated by consideration of the balance in the remaining immune cell subsets that have a wide spectrum of radiation sensitivities depending on their (1) lineage, (2) maturity, and (3) activation status (42). In brief, B cells and naive T helper (Th) cells are considered quite radiation sensitive whereas T memory cells, natural killer T cells, and Tregs are more on the resistant end of the spectrum (43–45). This relates in large part to a cell’s propensity to undergo apoptosis, which can drastically change as a result of activation (46, 47). Lineage recovery will also play its part in determining how the immune balance evolves in the aftermath of radiation treatment.

Remarkably, despite this layer of added sophistication, relatively crude values like the ratio of lymphocytes to granulocytes and/or monocytes can correlate with outcome. This may simply be a reflection of the general immune fitness of the patient, but may be more than that. In extreme cases, soaring granulocyte levels can be taken as a sign of bad prognosis, associated with enhanced metastasis and immune suppression through the development of myeloid-associated suppressor cells (48), which can readily be induced following either WBI or local RT. Radiation-induced myeloid cell activation can occur in the absence of tumor, but tumors can also release large amounts of myeloid growth factors, with or without RT (49–54). Such an induction of myeloid cells, post-RT is therefore an alternative mechanism to lymphodepletion as a cause of enhanced tumor growth and metastasis and targeting this can improve response to RT in preclinical models, although there is little evidence that this can result in regression and cure. Infections are another possible reason for a switch in immune balance from a lymphoid to more of a myeloid composition.

An optimist might look at this picture and suggest that within a certain immune context antitumor immune responses are ongoing, and that RT to the primary could enhance them, whereas a pessimist might point to the lack of clinical evidence for the immune system contributing to tumor cures in RT patients. It may turn out that both are correct, and that lymphocyte and myeloid cell involvement are simply two sides of the same coin.

## Does Successful RT Draw from the Immune System and *Vice Versa*?

In the 1970s, investigators at the MD Anderson Cancer Center performed a series of elegant experiments on an immunogenic 3-methylcholanthrene-induced fibrosarcoma model in C3H mice and illustrated that the curative success of local RT could clearly benefit from a healthy host immune status (55–57). For instance, the (local) radiation dose required to control 50% of irradiated tumors (TCD50) was increased about twofold if mice had previously been rendered incapable of mounting a T cell immune response through the classical depletion approach of adult thymectomy followed by lethal WBI and bone marrow rescue (58). This difference in dose is huge and the effect is made all the more dramatic by the finding that this normally non-metastatic tumor formed metastasis in 66% of the T cell-depleted mice, indicating the power of immunity in their elimination. Finally, in this study, only immune competent mice were able to develop immunological memory after radiation-induced tumor cure, demonstrating a lasting ability to reject subsequent tumor inocula. The authors reported considerable extra heterogeneity suggesting variability in the immune involvement in RT-induced cures in the form of a flatter probit curve for cure in intact mice compared with T-cell-depleted mice. It is worth noting that this model of T cell depletion by thymectomy has a natural tendency to develop autoimmunity due to preferential depletion of natural Treg. For example, in 1973, Penhale et al. reported that adult thymectomy of normal rats followed by five rounds of biweekly sublethal WBI (5 rad × 200 rad) produced autoimmune thyroiditis and type 1 diabetes (59). The importance of the Treg axis will be discussed below.

Experiments of the nature described above raise questions as to why immunogenic tumors grow in the first place. In fact, over 45 years ago, evidence was mounting that many human tumors contain tumor-specific antigens that can elicit host responses, but by and large clinically relevant immunity failed to surface (60). Many tumor escape mechanisms have been postulated, but one of the most powerful may simply be progressive tumor growth that overwhelms the response to even highly immunogenic tumors (56, 57). It may therefore be, in part, a numbers game and we know that RT is able to slow tumor growth and decrease the tumor burden, perhaps to immunologically manageable proportions, which raises the question as to what is manageable. According to Kaplan (61), immune eradication of 1% of a tumor may already translate into long-term survival benefits assuming that RT has taken care of the other 99%. The effectiveness of immune involvement in preclinical models can be estimated in terms of radiation dose. For example, for an immunogenic murine tumor, Suit and Kastelan (55) approximated that the immune system contributed a radiation dose to the equivalent of killing a few 100 cells, though, that doesn’t seem like much. However, one has to remember, first that the potency of the immune system can vary hugely and, second that dramatic immune-mediated regressions do occasionally occur. Immunity can also work against us when a multitude of suppressor mechanisms are engaged. In the immunogenic fibrosarcoma model used by Stone et al. (58), for instance, immunity is generated soon after tumor cell injection but is rapidly and strongly suppressed, initially by tumor-specific T cells and later by non-specific myeloid suppressor cells that finally shut down the whole immune system (62). What is clear is that RT, in the complexity of the irradiated host-tumor relationship, is more than a killer in a numbers game as suggested by classical target theory.

Another question raised by these experiments is whether RT induces a special form of “immunogenic cell death,” and if so, does this bestow RT with properties that sets it apart from other treatment options when it comes to complementing IT. Not surprisingly, for immunogenic tumors, removal of the primary tumor burden, by *any* means, is likely to lead to resurgence of a demonstrable tumor immune state and in that sense surgical removal of tumor can have a similar effect as “curative” RT. Photodynamic therapy seems to be especially powerful in this regard. There are not many examples where direct comparisons have been made between modalities, but Crile and Deodhar reported that RT of a Lewis fibrosarcoma in the footpad resulted in better control of metastasis than amputation (63). In any case, removal of the primary may do more than decrease the tumor burden. It may liberate the immune system. This is, in part, because innate or “natural” immune mechanisms differ from adaptive ones in possessing little by way of immunological memory, and natural Treg cells actually seem to fall into this category (64). Therefore, the removal of a tumor is likely to get rid off most if not all suppressor mechanisms while tumor-specific memory will remain, i.e., tilting the immune balance toward immunity. The timing of tumor removal relative to the state of the immune system will influence the outcome of such interventions, irrespective of the modality. There are other factors that may come into play, such as the rate of loss and/or prolonged release of tumor antigens, changes in tumor immunogenicity possibly associated with oxidative stress and the involvement of draining nodes, all of which are likely modality-specific and possibly give RT an edge over other therapies.

Like RT, surgery has been shown to both enhance and inhibit the number and the growth rate of secondary lesions. In their exceptional review on the subject, Demicheli et al. (65) noted that effects of primary tumors on those at distant sites were observed by Ehrlich and Apolant over a century ago. Apparently, a second inoculum of a rat sarcoma grew more slowly than the primary, a phenomenon for which Bashford and colleagues, in 1907, coined the term “concomitant immunity,” assuming involvement of the immune system (66). This idea, though, fell out of favor in the 1980s when Gorelik et al. showed that it could happen in immune-deprived animals and concluded that the mechanisms were different for immunogenic and non-immunogenic tumors (67). Prehn (68) postulated that a tumor behaved like an integrated organ liberating systemic growth-inhibiting and growth-facilitating factors, some of which were later identified by Folkman as angiogenesis inhibitors (69).

In the field of radiobiology, Mole (70) had introduced the term abscopal to describe effects “at a distance from the irradiated volume but within the same organism.” Mole in fact was discussing the interdependency of normal tissue systems responding to WBI, with no reference to cancer or immunity, but its use has since been extended to include RT of cancer and is often assumed to have an immune mechanism. Given that there are several excellent recent reviews dealing with abscopal effects in RT (71, 72), we will not go into the topic here, only to note that there seems to be more than one mechanism at play—depending on the system. Adaptive immunity may be involved, or not. To that end, Demaria et al. elegantly showed a tumor-specific immune abscopal effect of RT, whereas Camphausen’s team demonstrated abscopal effects that were not tumor-antigen specific (73, 74). Of interest in this context is a study by Hoch-Ligeti (75) where skin irradiation with soft X-rays decreased the incidence of chemically induced liver tumors. Whether it is normal tissue or tumors that are being exposed, there is no question as to RTs ability to drive many systemic forces, including cytokines, chemokines, acute phase reactants, and innate immune cells. These will influence events locally as well as at a distance and potentially engage antitumor immunity, angiogenic networks, hormones, or any other factors that can affect the growth of metastases. Clearly, tumor growth can wax and wane over time, as can the mechanisms that are involved, and our understanding of these processes are of tremendous value for the progress of combined RT and IT.

## Difficulties in Modeling Human Tumor Immunity

As described, most of the experimentation done in the 1970s used immunogenic transplantable tumors. It rapidly became obvious that often a relatively high number of tumor cells (10^3^–10^5^) had to be injected to get growth in 50% of mice (TD50). Nowadays, this is commonly explained by the low frequency of cancer stem cells, but at that time possible involvement of the immune system was considered and is still possible. In 1966, Klein had observed a tumor immune escape mechanism that was the opposite of that due to large tumor inocula (76). “Sneaking through” was defined as preferential take of small tumor inocula that exceeded what was seen in medium sized inocula, and more similar to large inocula. This was regarded as a possibly important mechanism by which tumors might subvert host defenses early in the development of the cancer. “Sneaking through” appeared to be a T-cell dependent phenomenon (77), analogous to the process of low-zone tolerance induction (78, 79) mediated by suppressor T cells (Ts) (80). In fact, both low and high inocula were found to induce immunological tolerance mediated by Ts cells, with the high inocula additionally inducing non-specific myeloid suppressor cells (81). Ironically, most investigators to this day utilize intermediate sizes of inocula that generate the best level of immunity to begin with. This, of course, will have implications for the responses that emerge after tumor RT because they relate to the state of immunity that exists at that point in time, transitioning rapidly to suppression as the tumor grows. We know of no studies that have looked at how existing tolerance affects the tumor response to RT.

In the mid-1970s, the relevance of chemically and virus-induced murine cancer models to the human condition was heavily criticized on the basis of their high immunogenicity. Perhaps one of the most vocal opponents was H. B. Hewitt from the Graylab (UK), who performed “isotransplants of 27 different tumours (leukaemias, sarcomata, carcinomata), all of strictly spontaneous origin in low cancer mouse strains… (showing that they) … revealed no evidence of tumour immunogenicity,” and concluded that “practically all animal data … entail artefactual immunity associated with viral or chemical induction” (82). This was a damning indictment of the field and, sadly, basically stalled further research. As far as RT is concerned, if the lack of immunogenicity was true, the immune system might end up not adding much efficacy (83). However, it should be noted in Hewitt’s study, that “for 7 randomly selected tumours, prior ‘immunization’ of recipients with homologous, lethally irradiated cells increased” tumor take. Since the generation of tumor immunity is highly dependent on the number of tumor cells injected (81), and because immunity can be a two-edged sword capable of both enhancing and suppressing tumor growth, it seems possible that tumor-specific responses did exist but could not be demonstrated in Hewitt’s model and under those conditions.

## Human Tumor Immunogenicity

The concept that human tumors had poor immunogenicity and little effect on the response to RT lingered until very recently even though it had become possible long ago to isolate leukocytes from cancer patients and clearly show they responded specifically to their own tumor *in vitro* (84–86).

Remarkably, DNA deep sequencing of human tumors has now revealed mutational signatures that can be linked to smoking and other harmful chemical exposures, UV radiation, viruses, and age. In many cases, these mutations may even be predicted to result in MHC-restricted neoantigens (87, 88). Formerly, “immunogenic” tumors used to be defined by a low but detectable tendency for spontaneous regression, as in melanoma. Then they were defined by activity when used as an irradiated vaccine, then by responding to high dose interleukin-2 (IL-2), as in kidney cancer. Now, the response to checkpoint inhibition has extended the list of human immunogenic tumors to include Merkel cell, esophageal, Hodgkin’s, and lung cancer. In fact, chemical cancer induction following harmful exposures goes back to observations of skin cancer of the scrotum among British chimney sweeps in 1775, viral induction by Rous in 1911, UV radiation induction by Findlay in 1928, and ionizing radiation by Muller in 1927 (11, 89–91). In a sense, we have come full circle, back to known causes of cancer and the spectrum of genetic mutations that are involved. These may drive the disease but may also hold the key for an immunological cure. In many cases, for chemically induced tumors the neoantigens may be unique. However, the fact that virus-induced cancers have actually a low mutational load but still respond to checkpoint inhibitor therapy similar to chemically induced forms (88) suggests that the number of mutations is not the be all and end all. Certainly, it is tempting to think that the reason why human papilloma virus+ head and neck tumors respond well to RT lies in their immunogenicity.

## Are Tumor-Infiltrating T Cells Exhausted?

In toto, the literature indicates that in most immunogenic tumor models, CD8+ T cells are an absolute requirement for regression, with varying “help” from CD4+ T cells, macrophages, and other immune compartments. Although not all tumor models behave the same way, this general finding is in keeping with the observations that in many human tumors the presence of CD8+ lymphocytes is associated with better prognosis. Many studies have attempted to correlate immune infiltrates with outcome with variable degrees of success.

The idea that intratumoral T cells might be exhausted became a school of thought in the 1980s when it was shown that potency could be restored by a few days of *in vitro* culture (85, 86). In fact, “exhausted” T cells probably mark many chronic conditions, including chronic infection. In cancer, they express high levels of inhibitory receptors, including programmed cell death 1 (PD-1), cytotoxic T-lymphocyte-associated protein 4 (CTLA-4), T-cell immunoglobulin mucin-3, and lymphocyte-activation gene 3, as well as showing impaired production of effector cytokines, such as IL-2, tumor necrosis factor alpha (TNF-α), and interferon gamma (92). They are void of effector functions, but these can be restored. This is reminiscent of the temporary loss of effector T cells seen in the spleen and organs from fibrosarcoma-bearing mice that had been successfully treated with *C. parvum* (93). In fact, tumor-specific T cell memory was retained in these mice, which became apparent when these cells effectively caused tumor regression in an adoptive transfer model, even though they had previously lost effector activity—a phenomenon that was called immunologic amnesia. Effector cell activity could also be restored during *in vitro* culture in T cell growth factor (IL-2). It seems reasonable to suggest that the immune system attempts to dampen chronic inflammatory states, including cancer, either through T regulatory cells or through directly blocking effector T cell function, and that the latter can be a result of the dialog between M2 macrophages and T cells as well as altered metabolism (94). The good news is that these roadblocks can be lifted, for example by targeting CTLA-4 or PD-1/programmed death-ligand 1 (PD-L1), respectively, allowing T cell memory to restore functional antitumor activity.

## Danger and the Chance to Add Insult to Injury

The logic for the use of radiation as an adjuvant to enhance antitumor immune responses is rather clearer now than it was in the 1900s, as fundamental immunological theories came together. The original self/non-self paradigm (95)^4^ and the concept of recognition of pathogen-associated molecular patterns (PAMPs) (96) explain how we detect a pathogenic threat, but fall short on explaining responses originating from within our own (damaged) tissues. The missing piece of the puzzle emerged in 1994 when Matzinger introduced the Danger theory that accommodated immune responses to damaged tissues through recognition of damage-associated molecular patterns, much as we can respond to PAMPs (97). Binding to common pattern recognition receptors culminates in inflammation with activation of signaling pathways such as nuclear factor kappa B, activator protein 1, and interferon regulatory factors, with type I interferon activation emerging as a possibly critical path toward radiation-induced tumor immunity (98–101). The possibility that radiation-damaged cells and tissues send out such danger signals to the immune system was outlined by McBride in the Failla Memorial Lecture at the International Congress of Radiation Research in 2003 (47). There is now considerable evidence supporting the idea that tissue irradiation feeds into down-stream immune effector pathways, even if involvement of specific toll-like receptors remains uncertain (102). Ultimately, one would expect increased immune recognition—autoimmunity or tumor immunity. Our ability to detect a rise in tumor-specific T cells in cancer patients as they go through RT certainly adds validity to this concept (103).

## Radiation, Inflammation, and Autoimmunity

There is a large body of work on radiation and autoimmunity, starting in the late 1990’, and earlier. The details of these studies are discussed elsewhere (42, 104) but for the purpose of this historical journey and considering the relevance to tumor immunology it is worth outlining the main findings and concepts here: perhaps the most striking of which is that tissue irradiation is able to both cause autoimmunity as well as suppress it.

In their most basic form tissue responses to RT can be described as *bona fide* inflammatory reactions that are driven by the extent of cell death and tissue damage. The release of danger signals, chemokines, and cytokines are doing their part to translate the situation to the immune system and attract inflammatory infiltrates to come into the irradiated area (98, 105–110). RT drives all of these steps, including a rise in MHC expression and costimulatory molecules that would—at least in theory—aid immune recognition and reactivity (111–116).

Indeed, radiation-induced immune responses to self within the context of normal tissues, i.e., autoimmunity, have been extensively reported. Anti-thyroid autoantibodies and thyroiditis following thyroid exposure (117, 118), multi-organ immune disease following total lymphoid irradiation (TLI) in mice (119), neoantigen formation, and morphea in the skin of irradiated breast cancer patients (120) are all strong indications for radiation-induced autoimmune disease, as are the T cell infiltrates seen in normal tissues of cancer patients and transplant recipients following irradiation and the local inflammatory reactions that ensue such as sialadenitis, interstitial pneumonitis, and alveolitis (121–125).

Ironically, this equation changes completely when the patient already has ongoing inflammation and/or autoimmune disease, i.e., when the immune balance has shifted in time and space to reach a new equilibrium. In such cases, WBI or TLI followed by autologous stem cell transplantation can rebalance T cell networks (126, 127) and alleviate for instance systemic lupus erythematosus and rheumatoid arthritis in humans or allergic encephalitis in mice (128–130). A similar case in point is the successful treatment of chronic, benign inflammatory conditions with local, low-dose radiation treatments (131–133).

## Radiation, Inflammation, and Tumor Immunity

Inflammation is a major component of human tumors and chronic inflammation tends to portend a bad prognosis. In fact, about 150 years ago, Virchow postulated that inflammation predisposes to cancer based on his observation that it often arose at sites of chronic inflammation and noted that inflammatory cells were often present in resected tumors. The involvement of infections and associated chronic inflammation as a common contributor to genetic instability, in addition to direct damage caused by chemicals, viruses, and radiation, is being resurrected as various forms of cancer are becoming closely associated with various microbes.

Apart from the pro-inflammatory effects mentioned above, RT has additional qualities that would feed into an inflammatory-tumor immunity axis. RTs ability to enhance the expression of the death receptor Fas on tumor cells is one such example, potentially sensitizing them to antigen-specific cytotoxic T cells and, ultimately, tumor rejection (134, 135). On the other hand, Fas is likely to play a role in radiation-induced lymphocyte death, and hence tolerance within the radiation field (136). RT can mature dendritic cells (DCs) so they can cross-present tumor antigens (137) and for a time at least RT can generate an immunologically permissive environment, something that seems to be especially amplified by hypofractionated doses (8). It is reasonable to suggest that hierarchical antigenic presentation by the tumor and by the DCs, may be affected during RT (138) making the case for altered T cell repertoires post-RT (115). The evidence that local RT dramatically alters the tumor-associated antigens that are released remains relatively limited, as is any proof that irradiated human tumors induce strong immunity, but there is growing evidence that “epitope spreading” is important for tumor rejection (139). What RT certainly can do, is improve the conditions for tumor immunity to occur, at least for immunogenic tumors.

While cancer RT is a pro-inflammatory stimulus, the term “inflammation” is totally inadequate to describe what is essentially a very complex set of pathological states that shift in time while progressing from what is blithely called “acute” to “chronic” states. Conditions that might help antitumor immunity can easily morph into ones that promote carcinogenesis, suppress immunity, and promote healing. And it may require drastic interventions to rebalance T cell networks, as in the likes of RT of autoimmune diseases (see above). One “natural” immune rebalancing act involves shifting the T cell equilibrium toward suppressor cells, i.e., Tregs, and this can happen following RT (45, 140–148). This concept that RT can drive the Treg lineage is discussed elsewhere (149) but one important point has to be emphasized here as it relates to a paradoxical observation made decades ago, namely that sublethal WBI can destroy Ts and as a result allow better tumor regression, presumably through an immune-mediated mechanism (46, 150–154). The obvious conclusion at the time was that Ts must be very sensitive to radiation. Though not wrong, it doesn’t mean that all Tregs are radiosensitive all the time. In fact, the WBI was only effective when given within a short time frame after tumor inoculation. Today we know that at any given time there are different subtypes of Tregs operating, each with the ability to alter their proliferative and/or activation status in response to a challenge and it is not difficult to see how that leads to fluctuations in radiation sensitivities (155). Given the focus on manipulating this T cell subset, it seems that there may be a use for RT in this context providing the correct timing can be found.

In a broader context, the outcome of RT with IT will heavily depend on the timing of exposures to these agents, i.e., the state of the immune system when radiation hits. This includes microenvironmental factors, especially the cytokine milieu that dictates trafficking, proliferation, activation, and differentiation of immune cells and tumor responses. Cytokine responses in the context of radiation damage have been extensively documented since the 1990s but to understand them in their full complexity can be daunting (156). Generally speaking, the cytokine picture that emerges after RT is one of dichotomy that reflects the two opposing forces of the immune system. In other words, RT affects not only the Tregs:Teffs immune balance but also shapes the ratios of Th1/Th2, M1/M2, and effector and suppressor cells of other lineages (157) making for an interesting future.

## Adverse Events

The normal tissue toxicities associated with conventional cancer radiotherapy are well-known, although the introduction of IMRT to deliver larger than normal dose per fraction has made treatment volume of growing importance, which is a change in the way radiobiological constraints are generally considered. IT is generally thought to be well tolerated in comparison with conventional cancer therapies (158), but the history of this also has changed. Cooley’s toxins, introduced at the end of the nineteenth century give expected “flu-like” symptoms similar to those of bacterial infections, as did *C. parvum* and BCG, that were used as immunological adjuvant cancer treatments since the 1960s.

By contrast, high dose IL-2 that was used for treatment of melanoma and kidney cancer is associated with significant morbidity. Common to many treatments, the incidence and severity of toxicities have decreased with the gain in experience that comes with use. IL-2 toxicity can manifest as multiple organ syndrome, most significantly involving the heart, lungs, kidneys, and central nervous system in capillary leak syndrome (CLS). As with most IT protocols, pharmacological intervention effectively manages the majority of adverse events, but fatalities have occurred. Treatment typically consists of supportive care with intravenous fluid, non-steroidal anti-inflammatory drugs, vasopressors (if needed), and other measures while awaiting spontaneous recovery. Since RT also causes CLS, the combination of these treatments would be expected to interact in at least a cumulative manner. Localization of the RT may minimize the consequences of the combination, but too few patients have been treated so far with this way for conclusions to be drawn and caution is advised. It should be noted that the dosage requirements for efficacy of IL-2 in the context of RT are also unknown and may have to be changed.

Toxicities associated with the combination of RT with adoptive T cell transfer are also currently unknown, but this topic is a likely one for future concern, especially when delivered with concurrent IL-2 administration. Currently, in the clinic, this IT approach most often employs *in vitro* expanded, tumor-specific T cells, or genetically modified populations that express tumor-directed TCRs or chimeric antigen receptors (CARs). The latter have an extracellular antigen-binding domain from the heavy and light chains of a monoclonal antibody that recognizes cell surface antigens linked to an intracellular signaling domain derived from the TCR complex, and can include one or more costimulatory molecules to enhance antitumor activity. On- and off-target toxicities are uncommon, but CARs treatment was fatal for several patients in a trial that ascribed the excessive toxicity, in this case cerebral edema, to the addition of fludarabine to the preconditioning regime (NCT02535364) (159). The concerns seem universal in that they revolve around the cytokine release syndrome that is observed shortly after T cell administration and additional symptoms similar to sepsis, with fever, tachycardia, vascular leak, oliguria, hypotension, neurotoxicity, and multi-organ failure (158). The mediators of the hemodynamic toxicities in these cytokine storms have yet to be fully identified but IL-6 and TNF-α may be the prime culprits, both of which can be generated by RT.

The advent of checkpoint blockade IT has unveiled a slightly different spectrum of toxicities. These have been called “immunerelated adverse events” (irAEs) and have focused attention on opportunistic autoimmune disorders (160). Depending on the target, the toxicities associated with checkpoint inhibition may vary, but there are elements in common. CTLA-4 counteracts CD28-mediated costimulation and induces an inhibitory program that stops T cell proliferation while driving Treg cells. As CTLA-4 plays a pivotal role in regulating tolerance to self-antigens, CTLA-4 blockade with ipilimumab or tremelimumab, can be understood as a lowering of the threshold for T cell activation and hence results in autoimmune damage of various organ systems. PD-1 is another member of the family of coinhibitory receptors (checkpoints) expressed on activated T cells. Interaction with its ligands PD-L1/B7-H1 and PD-L2/B7-DC on other cells delivers inhibitory signals to T cells. In general, over half of patients receiving approved checkpoint inhibitors experience a low grade irAE; serious adverse reactions are relatively rare, with <1% mortality (160), but the combination of checkpoint inhibitors is more toxic and RT would be expected to increase their incidence. Any organ system may be involved, but the most common are enterocolitis, hepatitis, dermatitis, thyroiditis, uveitis, neuropathy, pneumonitis, and endocrinopathy. A bitter lesson as to the power of the immunological synapse was learned from the disastrously trial of TGN1412, an anti-CD28 superagonist antibody that caused catastrophic organ failures in all subjects (161).

Cytotoxic T-lymphocyte-associated protein 4 blockade tends to compromise mucosal immunity in particular and overall drives a more severe toxicity profile than inhibitors of the PD-1/L1 axis. Data on PD-L1 targeting are less developed but 9% grades 3–4 toxic side effects have been reported (162). Though rare, cardiovascular toxicity has been reported and can lead to significant morbidity and mortality especially in cases of pre-existing pathologies (163, 164). Among the immune-related cardiac syndromes reported after anti-CTLA-4 and anti-PD-1 therapies are autoimmune myocarditis, cardiomyopathy, heart failure, cardiac fibrosis, and cardiac arrest, even more so if the agents are combined. Certainly, pharmacologic or genetic targeting of PD-1 in animal myocarditis models tell a cautionary tale. It seems that PD-1 is very important in protecting the heart against T cell-mediated toxicity that otherwise would translates into enhanced disease severity, rising troponin levels as well as infiltrating lymphocytes, macrophages, and neutrophils (165). PD-L1 suppression may not always be as devastating but the take-home message is that the PD-1–PD-L1/L2 axis is an important checkpoint for myocyte damage and cardiac pathologies (166–170). Increased atherosclerotic lesion development and inflammation are additional concerns (171). Interestingly, pneumonitis may not be as much of a problem during PD-L1 targeting as it is during PD-1 blockade as protection *via* PD-L2 remains intact in the former therapy (160).

Radiation therapy is pro-inflammatory and this is especially true at high dose per fraction. It is likely to increase the incidence of autoimmune reactions and, when combined with checkpoint inhibitors, more severe toxicities are to be expected. While it is reasonable to suggest that the toxicities may be greatest in the organs that receive substantial doses of RT, this may not be always the case as systemic responses are triggered. Apart from a few of studies on RT and IT of melanoma brain metastasis, with no obvious excess toxicity (172–174), the incidence of treatment toxicities to IT combinations remains largely unknown but with over 800 combined checkpoint inhibitor trials in the pipeline, and 100 in the context of RT (Table 1), it will be soon.

**Table 1:** **Radiotherapy–immunotherapy (IT) combination trials currently open**.^a^

Immune axis	Drug	Radiotherapy	Indication	Number of patients
Cytotoxic T-lymphocyte-associated protein 4 (CTLA-4)	Ipilimumab, tremelimumab	Hypofractionated stereotactic body radiation therapy (SBRT), stereotactic ablative body radiation therapy (SABR)	Metastatic melanoma, advanced malignancies (liver, lung, cervix)	400
Programmed cell death 1 (PD-1)	Pembrolizumab, nivolumab	Mostly hypofractionated SBRT, some SABR, chemoradiation, intensity-modulated radiotherapy (IMRT), stereotactic radiosurgery	Metastatic melanoma, liver, head and neck squamous cell carcinoma (SCCHN), metastatic breast cancer, small cell lung carcinoma (SCLC), non-small cell lung carcinoma (NSCLC), metastatic renal cell carcinoma (mRCC), glioblastoma multiforme, metastatic colorectal carcinoma (mCRC), pancreatic cancer, follicular non-Hodgkin’s lymphoma, bladder, endometrial cancer	4,253
Programmed death-ligand 1 (PD-L1)	Durvalumab, atezolizumab, or avelumab	Hypofractionated SBRT, some SABR, chemoradiation, IMRT	Metastatic non-small cell lung carcinoma (mNSCLC), SCCHN, metastatic Merkel cell, glioma, metastatic pancreatic cancer, esophogeal cancer	1,273
PD-1/PD-L1 + CTLA-4	Nivolumab + ipilimumab or durvalumab + tremelimumab	Hypofractionated external beam radiotherapy (EBRT), some SBRT, chemoradiation, yttrium Y-90 selective internal radiation therapy	Metastatic melanoma, SCLC, mNSCLC, mCRC, pancreatic cancer, liver mets, brain mets	1,017
Interleukin-2, toll-like receptor 7, recombinant human FMS-like tyrosine kinase 3 ligand, Poly-ICLC, OX-40, recombinant human granulocyte-macrophage colony-stimulating factor, transforming growth factor beta, IDO, fibronectin	Proleukin, imiquimod, CDX-301, hiltonol, MEDI6469, sargramostim, galunisertib, indoximod	Hypofractionated SBRT, SABR, chemoradiation, low-dose radiation therapy (RT)	Metastatic melanoma, mRCC, metastatic breast cancer, advanced NSCLC, hepatocellular cancer, lymphoma, rectal cancer, pediatric brain tumors	462
Therapeutic cancer vaccines	Autologous dendritic cell vaccine, peptide vaccine, sipuleucel-T, nelipepimut-S	Chemoradiation, IMRT, SABR, i.v. radium-223, standard of care RT before IT	Glioma, locally advanced esophageal cancer, NSCLC, metastatic castrate-resistant prostate cancer, high-risk breast cancer, pediatric glioma	774
Adoptive T cell transfer	Autologous T-cells	EBRT or chemoradiation	Esophageal cancer, nasopharyngeal cancer, glioma	223
Oncolytic virus and antibody tumor targeting	Adenovirus-mediated herpes simplex virus thymidine kinase + valacyclovir, herpes simplex virus type 1 G207, bavituximab (phosphatidylserine), oregovomab (CA125)	Chemoradiation, EBRT, hypofractionated SBRT	Pancreatic adenocarcinoma, localized prostate cancer, pediatric brain tumor, hepatocellular carcinoma	857

*^a^Source: https://clinicaltrials.gov/, date searched: January 31, 2017, search terms: radiation, PD-1, PD-L1, CTLA-4, radiotherapy, and immunotherapy*.

*Trials using immunotherapy that directly follows standard of care radiation treatment were included. Excluded were any trials that used radiation as a preconditioning regime prior to bone marrow transplantation or if radiotherapy was offered solely as a best supportive care option and not as a definite treatment option. Salvage radiotherapy after failed immunotherapy or vice versa was not included, neither was targeting CD20/CD19 nor EGFR in the context of radiation treatment*.

## Conclusion

It is easy to appreciate how RT can be a double-edge sword in the case of immune reactivity being a potential major benefit but increasing the risk of normal tissue complications. Such is the fundamental nature of this dilemma that it embodies one of the most challenging aspects of cancer therapy, namely how to affect cure while minimizing side effects. However, the other side of this argument is that a tumor is already a site of ongoing immune involvement, and hence, something that RT might alter in analogy to the radiation-induced suppression of already existing autoimmune diseases or chronic inflammation. An interesting and perhaps philosophical take on this comes from Drs. Prehn who suggested tumor and host evolve along a bell-shaped immune response curve reaching a perfect equilibrium at the top when immunity is most conducive to tumor growth and that basically any attempt at shifting this balance, be it through IT, RT, or otherwise, would inevitably alter the perfect “tumor-immune dance” and slow tumor growth (175, 176).

## Author Notes

The original units of radiation dose (rad, Roentgen, or Gy) were quoted as in the original literature. However, for the purpose of comparison, the following assumption was made: 1 Gy = 100 rad (rad) ≈ 100 R (Roentgen).

## Author Contribution

DS conceptualized and wrote this manuscript.

## Conflict of Interest Statement

The author declares that the research was conducted in the absence of any commercial or financial relationships that could be construed as a potential conflict of interest.

## Footnotes

^1^http://post.queensu.ca/~forsdyke/murphy01.htm.

^2^Oral History Collection, American Philosophical Society.

^3^https://www.nobelprize.org/nobel_prizes/medicine/laureates/1908/mechnikov-lecture.html.

^4^https://www.nobelprize.org/nobel_prizes/medicine/laureates/1960/burnet-lecture.pdf.

## Abbreviations

AdV-tk, adenovirus-mediated herpes simplex virus thymidine kinase; AP1, activator protein 1; BCG, bacillus Calmette–Guérin; CARs, chimeric antigen receptors; CLS, capillary leak syndrome; CRS, cytokine release syndrome; CTLA-4, cytotoxic T-lymphocyte-associated protein 4; CRC, colorectal carcinoma; CSF, colony-stimulating factor; CV, cardiovascular; DAMPs, damage-associated molecular patterns; DCs, dendritic cells; EBRT, external beam radiotherapy; GBM, glioblastoma multiforme; Gy, radiation unit; HPV, human papilloma virus; HSV-1, herpes simplex virus type 1; MHC, major histocompatibility complex; IDO, indoleamine 2,3-dioxygenase; irAEs, immune-related adverse events; IFN-γ, interferon gamma; IL-2, interleukin-2; IMRT, intensity-modulated radiotherapy; IRFs, interferon regulatory factors; IT, immunotherapy; LAG-3, lymphocyte-activation gene 3; mCRC, metastatic colorectal carcinoma; mNSCLC, metastatic non-small cell lung carcinoma; mRCC, metastatic renal cell carcinoma; NF-κB, nuclear factor kappa B; NKT, natural killer T cells; NSCLC, non-small cell lung carcinoma; OX-40, tumor necrosis factor receptor superfamily, member 4; PAMPs, pathogen-associated molecular patterns; PD-1, programmed cell death 1; PD-L1, programmed death-ligand 1; Poly-ICLC, polyinosinic-polycytidylic acid, poly-l-lysine double-stranded RNA; rhuFLT3L, recombinant human FMS-like tyrosine kinase 3 ligand; rhuGM-CSF, recombinant human granulocyte-macrophage colony-stimulating factor; RT, radiation therapy; SABR, stereotactic ablative body radiation therapy; SCCHN, head and neck squamous cell carcinoma; SCLC, small cell lung carcinoma; SBRT, stereotactic body radiation therapy; SRS, stereotactic radiosurgery; TCD50, 50% tumor control dose (Gy); TCGF, T cell growth factor; TD50, 50% tumor take (number of cells injected); Teff, T effector cells; TGFβ, transforming growth factor beta; Th, T helper cells; TIM-3, T-cell immunoglobulin mucin-3; TLI, total lymphoid irradiation; TLR7, toll-like receptor 7; TNF-α, tumor necrosis factor alpha; T_reg_, T regulatory (suppressor) cells (Ts); WBI, whole body irradiation; Y90 SIRT, yttrium Y-90 selective internal radiation therapy.
